# Exciting times for inhibition: GABAergic synaptic transmission in dentate gyrus interneuron networks

**DOI:** 10.3389/fncir.2015.00013

**Published:** 2015-03-18

**Authors:** Srikanth Ramaswamy

**Affiliations:** Blue Brain Project, Brain Mind Institute, Ecole Polytechnique Fédérale de LausanneLausanne, Switzerland

**Keywords:** hippocampus, dentate gyrus, basket cells, synaptic transmission, inhibition, parvalbumin, cholecystokinin, somatostatin

Intricate cognitive functions supported by the cerebral cortex endow mammals with a profound ability to adapt to a constantly changing environment. These sophisticated functions arise as a result of complex synaptic interactions between a myriad of neurons, modulating their structure and function. Decades of studies have enriched our current knowledge on the fundamental principles of neuronal and synaptic organization in the cortex (Shepherd, 2004). Much of this knowledge comes from exhaustive studies of glutamatergic principal cells across different cortical regions (for review see Spruston, 2008). However, our understanding of the daunting diversity of inhibitory GABAergic interneurons and their function remains far from complete (for reviews see Freund and Buzsáki, 1996; Markram et al., 2004).

GABAergic inhibition in the cortex choreographs concerted synchronous and oscillatory activity. A noteworthy amount of previous work has increased our understanding on the physiology of synaptic interactions between GABAergic interneurons and pyramidal neurons across several cortical areas and regions (Somogyi et al., 1998; Gupta et al., 2000). Recent experimental advances in synaptic physiology have furthered direct access of networks of morphologically and molecularly distinct populations of GABAergic interneurons in the cortex (Pfeffer et al., 2013), and have begun to unravel intricate connectivity principles in networks of GABAergic interneurons.

In a recent article published in *The Journal of Neuroscience*, Savanthrapadian et al. (2014) undertook paired recordings of morphologically identified GABAergic interneurons in adult rodent dentate gyrus (DG) slices *in vitro* to unravel sophisticated local synaptic interactions. As an integral part of the hippocampal formation, the DG relays information from the entorhinal cortex to hippocampal CA3. The authors dissected inhibitory monosynaptic connections in the DG microcircuitry between parvalbumin (PV) expressing perisomatic targeting basket cells (BC), cholecystokinin (CCK) containing interneurons associated with the hilar commissural associational path (HICAP), and somatostatin expressing hilar perforant path associated (HIPP) interneurons. *Post hoc* morphological analyses of biocytin filled neurons revealed intricate patterns of local axonal and dendritic arbourization, hypothesizing plausible domains that specifically govern the spatio-temporal precision of GABAergic inhibition.

Subsequently, the authors mapped out the local synaptic anatomy and physiology of homologous and heterologous GABAergic interneuron networks through electrophysiological and optophysiological techniques. The authors discovered that the specific combination of pre-post morphologies was critical in determining the time-course of inhibition. Among examined homologous connections, HIPP–HIPP interactions occurred with the least probability, followed by a slightly higher occurrence of BC–BC pairs, whereas HICAP–HICAP interneuron pairs interacted most prevalently (see Figure 1). The physiology of homologous synaptic interactions displayed slow inhibition for HIPP–HIPP connections, while HICAP–HICAP synapses demonstrated intermediate levels of inhibition, followed by fast inhibitory transmission in BC–BC connections. Furthermore, neurotransmitter release in these homologous inhibitory networks was marked by exactness in short-term synaptic dynamics. The short-term dynamic properties of inhibitory signaling in homologous networks evinced multiple-pulse facilitation in HICAP–HICAP connections, while biphasic modulation was observed at HIPP–HIPP interactions, and pronounced depression at BC–BC synapses. In the case of heterologous connections, although the prevalence of connectivity of HICAP–BC and HIPP–BC interactions was marginally higher than homologous BC-BC connections, their physiology was measured to be unreliable and slow. The authors then directly recruited axon terminals of SOM containing HIPP interneurons expressing channelrhodopsin by stimulating the perforant pathway (PP) and simultaneously recorded the post-synaptic response in PV-positive BCs. Weak, unreliable and slow time course of inhibition originating from the dendrites of SOM containing HIPP interneurons remarkably regulated action potential (AP) kinetics by significantly diminishing their discharge while fine-tuning their temporal precision.

**Figure 1:**
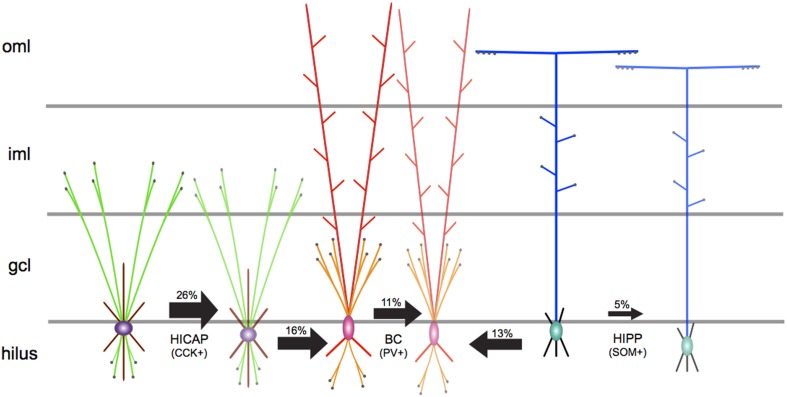
**A schematic representation of the synaptic network underlying principal GABAergic interneuron types in the DG microcircuitry**. The directionality of arrows depicts unidirectional inhibitory synaptic connections between identified pre-post interneuron types. The thickness of arrows depicts the strength of inhibitory connections. The measured connection probability (%) is indicated above each arrow. Axons are shown in green for HICAP, orange for BC, and blue for HIPP interneuron types. Terminal boutons are shown as black filled circles. Soma and dendrites are, respectively, shown in purple and maroon for HICAP, pink and red for BC, and green and black for HIPP interneurons. Gray lines depict layer boundaries (not to scale). oml, outer molecular layer; iml, inner molecular layer; gcl, granule cell layer.

The DG microcircuitry displays a specific laminar organization of afferents, which include the PP from the lateral and medial entorhinal cortex and commissural-association (CA) path from ipsilateral and contralateral hippocampus (for reviews see Förster et al., 2006; Amaral et al., 2007). BCs and HICAPs are innervated from excitatory PP afferents, the CA pathway, and granule cells (GC) and mediate feedforward or feedback inhibition (Sambandan et al., 2010). On the contrary, HIPPs are primarily innervated by mainly GC inputs and contribute to feedback inhibition (Hosp et al., 2014). The authors provide implications on the functional roles of these DG interneurons in pattern separation (Leutgeb et al., 2007), and suggest that BCs and HICAPs could be directly enlisted to provide rapid perisomatic and proximal dendritic inhibition onto GCs and interneurons, respectively. The authors suggest that the organization of DG microcircuitry indicates a rearrangement from perisomatic to distal dendritic signaling during a single PP-mediated wave of excitation. Furthermore, the authors identify functional similarities in signaling changeover from perisomatic to distal dendritic inhibition in DG microcircuitry with previous reports in hippocampal CA1 (Pouille and Scanziani, 2004).

Could dendritic inhibition be so positioned as to decisively alter the collective activity of local synaptic networks of GABAergic interneurons and control global information flow in the DG microcircuitry? This line of thought has been previously corroborated in the adult rodent somatosensory cortex by Ali and Thomson (2008). Indeed, the honing of the temporal precision of APs appears to be a design principle that endows specific GABAergic cell types to exert varying inhibitory influence encompassing a dynamic spectrum of local inhibition. The current study by Savanthrapadian et al. (2014) not only reinforces the premise of distinct GABAergic interneurons contributing to a continuous inhibition spectrum (fast, intermediate, and slow) but also provides preliminary evidence hinting toward a universal strategy of inhibitory synaptic transmission across different cortical areas and regions.

The authors have presented detailed analyses and results of their work, however, several open questions remain to be addressed. Previous work has conclusively demonstrated that GABAergic interneurons, particularly PV containing basket cells in neocortex or the hippocampal formation interact through a combination of chemical and electrical synapses (Bartos et al., 2007; Hu et al., 2014). The set of experiments presented by the authors does not address gap-junctional coupling between GABAergic interneurons in the DG, which renders itself as a future avenue to pursue.

Intriguingly, the authors report a striking lack of HIPP–HICAP connections, although there is a likelihood of putative HIPP–HICAP connections arising by virtue of axo-dendritic proximity. The authors propose a functional role for this apparent lack of HIPP–HICAP connections, postulating that inhibition of proximal and distal dendritic domains are not competitive but rather complementary in a spatio-temporal fashion. Indeed, further quantitative investigations are warranted for example, to examine whether possible chemo-repellant mechanisms at play during various stages of development in the hippocampal formation cause a selective eschewal in the formation of functional synapses between specific populations of GABAergic interneurons. Groundbreaking experiments that couple single cell optogenetic interrogations with paired whole-cell recordings *in vivo* from genetically defined GABAergic neurons will enable the characterization of inhibitory synaptic transmission in intact DG microcircuitry (see Pala and Petersen, 2015, for recent work in rodent neocortex).

The study in consideration endeavored to identify some of the mechanisms underlying the physiology of inhibition among identified interneurons in DG microcircuitry. Taken together, morphological and physiological analyses indicate that the number and location of inhibitory synapses, their conductance kinetics, as well as dendritic properties deliberate the efficacy and time course on somatic inhibition predominantly mediated by ionotropic GABA_A_ receptors. The authors speculate that putative anatomical synaptic contacts in HICAP–HICAP and HIPP–HIPP interneuron connections are fewer than at BC–BC connections. Indeed, the number of putative synaptic contacts is very likely an underestimate given that neuronal arbors are predisposed to severing attributed to slicing artifacts. In their study, the authors have not addressed connection specific binomial analyses of the voltage clamp synaptic recordings. Complementary anatomical and binomial analyses of GABAergic synaptic connections could reveal informative correlations between putative synapses and functional release sites, identifying connection specific mechanisms of unitary or multi-vesicular synaptic release as shown previously in neocortex (Gupta et al., 2000).

It is known that a non-linear density distribution of the *I_h_* current in dendrites of neocortical pyramidal neurons controls the time course of inhibitory synaptic transmission (Williams and Stuart, 2003; Silberberg and Markram, 2007). Although not directly within the scope of the current study, an essential next step is a characterization of the kinetics and density distributions of the zoo of ion channels expressed in specific GABAergic interneuron types. Such advances are imperative to develop a comprehensive picture of inhibitory synaptic transmission in DG microcircuitry.

The authors have put together a novel data resource in characterizing the inhibitory synaptic network underlying DG microcircuitry and postulate computational roles of GABAergic interneurons in the hippocampal formation. The data set provides an overview of the organizing principles of elementary building blocks constituting the local DG microcircuit—receptors, synapses, neurons, and their interactions. Further experimental work is imperative to increase our understanding of how a myriad of neuromodulators regulate these building blocks to assemble an integrated view of the function of DG microcircuitry in the emergence of different behavioral states. Such data sets complemented by *in vivo* mapping of long-range efferents could facilitate scaffold digital reconstructions of the DG microcircuit *in silico* to develop a unifying picture of its anatomy and physiology in brain function and dysfunction.

## Conflict of interest statement

The author declares that the research was conducted in the absence of any commercial or financial relationships that could be construed as a potential conflict of interest.
